# Association between dietary patterns and existing natural teeth in Chinese elderly: a national community-based study

**DOI:** 10.3389/fnut.2025.1549181

**Published:** 2025-03-13

**Authors:** Deng Huang, Pan Ding, Chao Lin, Liuhong Tian, Xiaodan Kuang, Jiaming Fang, Shulei Chen, Rongxiu Ding

**Affiliations:** ^1^Department of Quality Management, Wenzhou People's Hospital, Wenzhou, Zhejiang, China; ^2^School of Mental Health, Wenzhou Medical University, Wenzhou, Zhejiang, China; ^3^Department of Clinical Laboratory, Key Laboratory of Clinical Laboratory Diagnosis and Translational Research of Zhejiang Province, the First Affiliated Hospital of Wenzhou Medical University, Wenzhou, Zhejiang, China; ^4^Department of Preventive Medicine, School of Public Health, Wenzhou Medical University, Wenzhou, China; ^5^School and Hospital of Stomatology, Wenzhou Medical University, Wenzhou, Zhejiang, China

**Keywords:** protein diet, sugar-salt diet, anti-inflammatory diet, existing natural teeth, BMI, Chinese elderly

## Abstract

**Objective:**

To promote healthy aging, we aimed to evaluate the independent and joint effects of protein, sugar-salt, and anti-inflammatory diets on existing natural teeth among the Chinese elderly, and further explore the mediating role of body mass index (BMI).

**Methods:**

Based on the 2017–2019 Chinese Longitudinal Healthy Longevity Survey (CLHLS), 11,608 participants aged 65 and above were recruited in this cross-sectional study. Protein, sugar-salt, and anti-inflammatory diets were measured via a simplified 13-item dietary frequency questionnaire (dietary frequency around age 60). Restricted cubic spline and multiple linear regression analyses evaluated associations between dietary patterns and existing natural teeth, and mediation analysis explored BMI’s role.

**Results:**

Protein and anti-inflammatory diets were positively and linearly associated with existing natural teeth, while sugar-salt diets were negatively and linearly associated. Compared with the low dietary patterns (Q1), high protein and anti-inflammatory diets (Q4) were associated with a higher number of teeth (*β*: 1.70, 95%CI: 1.14, 2.25; *β*: 1.98, 95%CI: 1.45, 2.51, respectively; and 28% and 38% risk decreased for fewer than 20 teeth, respectively), whereas high sugar-salt diets had the lowest number (*β*: −1.14, 95%CI: −1.61, −0.67; 44% risk increased for fewer than 20 teeth). We further found a joint effect of low protein, high sugar-salt, and low anti-inflammatory diets on existing natural teeth (*β*: −1.97, 95% CI: −2.61, −1.33). Moreover, BMI mediated 10.88%, 19.69%, and 10.74% of the effects of the protein, sugar-salt, and anti-inflammatory diets with existing teeth, respectively.

**Conclusion:**

Promoting high protein and anti-inflammatory diets and reducing sugar-salt intake in elderly individuals may reduce tooth loss, possibly partly mediated through overweight or obesity.

## 1 Introduction

With the acceleration of the global aging process, the health of the elderly population is increasingly receiving public and social attention. According to the 2023 National Bulletin on the Development of the Aging Career (1), China has become one of the most seriously aging countries in the world, with more than 290 million elderly people aged 60 years and older (21.1%) and more than 210 million people aged 65 years and older (15.4%). Tooth loss is a prevalent issue among older adults and represents a key marker of oral health that significantly impacts systemic health, nutrition, and quality of life (2). The existing natural teeth in the elderly are associated with better masticatory function, improved dietary intake, and overall well-being (3). Conversely, extensive tooth loss is linked to malnutrition, cognitive decline (4), and increased mortality risk (5). Understanding the factors that influence natural tooth retention is therefore essential for promoting healthy aging.

Dietary frequency, which is the number of times an individual consumes food per day or week, has received increasing attention in recent years as a pattern of dietary behavior that can be easily monitored and intervened (6, 7). The implementation of healthy dietary patterns is a potential global health strategy to mitigate health problems in old age that occur with age (8). Previous studies have demonstrated the neuroprotective effects of polyphenol-rich plant-based diets, which may prevent or ameliorate diseases such as cardiovascular disease (9), depression (10), cognitive impairment (11), and mortality (6); furthermore, Omega-3 fatty acids present in some foods such as fish, legumes, and nuts may be able to improve functional brain health and reduce the risk of dementia (12); and diets high in protein may reduce the risk of frailty in the elderly population (13). Although the effects of dietary patterns on physical and mental health have been extensively demonstrated in many studies, few studies have reported on the relationship between past dietary patterns and the number of existing teeth in older adults. On the one hand, while diets high in sugar and salt have long been recognized as a major contributor to dental caries (14), evidence linking them to tooth loss has yet to be confirmed. On the other hand, protein-rich diets and high anti-inflammatory diets have been shown to have a mitigating effect on chronic inflammation (e.g., periodontitis) (15, 16), but the health effects of protein diets versus anti-inflammatory diets on tooth loss have also been little explored. While these studies provide valuable insights, most have focused on isolated dietary components or single nutrients (e.g., minerals and vitamins in food) (17, 18). Moreover, they are often conducted in Western populations, where dietary habits, healthcare access, and lifestyle factors differ significantly from those in non-Western contexts. Few studies have comprehensively examined the joint effects of multiple dietary patterns on existing natural teeth, especially in Asian populations.

Furthermore, prior research has rarely accounted for the mediating role of metabolic factors such as body mass index (BMI), which links dietary patterns with oral health through systemic mechanisms (19–21). Investigating these pathways may provide deeper insights into the complex interplay between diet, metabolic health, and tooth retention.

Based on the Chinese Longitudinal Survey of Healthy Longevity (CLHLS) population aged 65 years and older, this study aimed to assess the association between protein diet, sugar-salt diet, and anti-inflammatory diet at around age 60 years and the existing natural teeth by comprehensively adjusting for general demographic characteristics, lifestyle behaviors, and health confounders. Meanwhile, we made several hypotheses: (a) There is a linear or non-linear association between dietary patterns and existing natural teeth. (b) There is a joint effect of multiple dietary patterns on existing natural teeth. (c) There is a mediating role of BMI in the association between dietary patterns and existing teeth. By comprehensively evaluating the study of the interaction of these dietary patterns and metabolic factors in a community-based elderly population in China, we pin our hopes on informing dietary recommendations and public health interventions to improve oral health and overall quality of life in the elderly population.

## 2 Methods

### 2.1 Study design and participants

Our participants were from the 7th wave (2017–2019) of the Chinese Longitudinal Healthy Longevity Survey (CLHLS). The CLHLS began in 1998 and was conducted by the Chinese Center for Disease Control and Prevention (CDC) using disproportionate and targeted sampling methods for the elderly population aged 65 years and older (22). The survey covered 22 provinces, autonomous regions, and municipalities directly under the central government, representing China’s diverse geographic regions, urban–rural differences, and levels of economic development (23). The questionnaire was led by Peking University and participated by academic institutions and researchers from multiple countries (24). The questionnaire covers general demographic characteristics, behaviors and lifestyles, social support and economic factors, and health status in old age (23), making it one of the largest and longest-tracking surveys on health and longevity in the world. CLHLS data were collected face-to-face by highly trained enumerators using standardized questionnaires and measurement tools, with a total of 113 thousand household visits. The questionnaire has a low non-response rate (4.85% per wave on average) (25), similar to large Western cohort studies, and is an important resource for global aging research.

**Figure 1:**
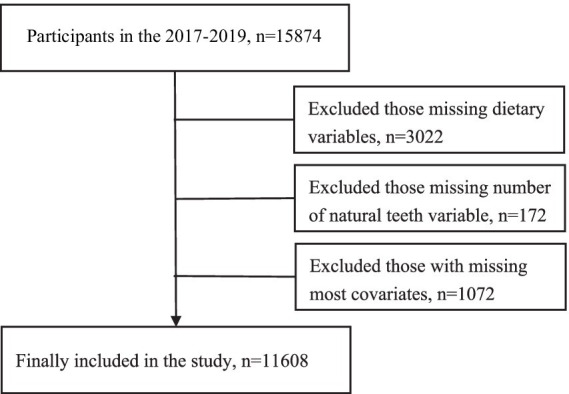
Flow chart of the study population.

Of the 15,874 participants surveyed from 2017 to 2019, we excluded 3,022 participants missing dietary variables, further excluded 172 participants missing the variable of existing natural teeth, and excluded 1,072 participants missing most of the covariates (lifestyle and health variables, etc.), ultimately including 11,608 participants (Figure 1). We compared dietary patterns and the existing natural teeth between included (*n* = 11,608) and excluded participants (*n* = 1,072), and the results showed no statistically significant differences in the two populations (*p* > 0.05; Supplementary Table S1). The CLHLS was approved by the research ethics committee of Peking University (IRB00001052-13074) and obtained informed consent from all participants.

### 2.2 Assessment of dietary patterns

Participants were asked about the frequency of the 13 foods they ate when they were about 60 years old (26), and meal frequency was categorized into five groups: “rarely or never,” “not monthly, but sometimes,” “at least once a month,” “at least once a week,” and “almost every day” (scored as 0 to 4). Based on previous studies (6, 27), we defined 5 protein-rich diets as protein diets (meat, fish, eggs, legume products, and dairy products, with a score range of 0–20) (27); pickled and salted foods and sugary foods as sugar-salt diets (with a score range of 0–8) (6); and anti-inflammatory diets that included 6 categories: garlic, nuts, mushrooms and algae, vitamins or nutraceuticals, medicinal plants, and tea (with a score range of 0–24) (27). In our study, this dietary frequency scale showed high internal consistency with a Cronbach’s alpha coefficient of 0.796. In addition, the main reason we used participants’ dietary frequency when they were around 60 years of age rather than their current dietary frequency was to avoid potential reverse causality. For further exploration of the joint effect, we divided the three dietary patterns (protein diet, sugar-salt diet, and anti-inflammatory diet) into two groups based on median and recombined them to obtain 8-categorical data.

### 2.3 Assessment of existing natural teeth

The existing natural teeth were self-reported by the participants and assessed by asking “How many natural teeth do you have (excluding dentures)” (28, 29). When assessing participants’ baseline characteristics, we categorized the existing natural teeth into four groups by quartiles (30): 0 (*n* = 3,695), 1–6 (*n* = 2,348), 7–20 (*n* = 3,090), and > 20 (*n* = 2,475). Previous evidence suggests that when the existing teeth are below 20 it affects the functional health and esthetics of the oral cavity (31, 32), in the secondary analysis we divided the natural existing teeth into two groups (<20, ≥20) to study the effect of dietary patterns on them.

### 2.4 Assessment of covariates

The selected covariates were based on previous literature (22, 23, 33) and expert opinion. In this study, we included general demographic characteristics: sex (male, female), age (<80, <90, ≥90), residence (city, town, rural), economic status (good, general, poor), education (illiterate, primary school, middle school or above), widowed (yes, no), self-reported quality of life (good, fair, poor); behavioral lifestyles: sleep duration (<7 h, 7–8 h, >8 h), drinking status (current, former, never), smoking status (current, former, never), current exercise (yes, no); health status: body type (underweight, normal, overweight, obese), self-rated health (good, fair, poor), cognitive function (normal, impaired), activities of daily living (normal, impaired), multimorbidity (yes, no), depression (yes, no).

Body Mass Index (BMI) was obtained by calculating the weight (kg) and square of height (m). Body type was categorized into four categories according to Chinese standards (34): underweight (<18.5 kg/m^2^), normal (18.5–23.9 kg/m^2^), overweight (24.0–27.9 kg/m^2^), and obese (≥28.0 kg/m^2^). Cognitive function was assessed based on a 25-item Chinese version of the Mini-Mental State Examination (CMMSE, total score of 30), with a score below 24 recognized as cognitively impaired (35). Activities of daily living (ADL) were assessed by a 6-item scale (bathing, dressing, toileting, indoor transferring, continence, and eating), with impaired ADL recognized when more than 1 item required assistance (24). We defined multimorbidity using conditions common to older adults, who were considered to have multimorbidity when they self-reported one or more of the following conditions (36): hypertension, diabetes mellitus, heart disease, bronchitis, emphysema, pneumonia, asthma, stroke or cerebrovascular disease, and arthritis. Depression was assessed by the 10-item Center for Epidemiological Studies Depression Scale (CES-D-10, total score of 30), with a score of more than 10 being recognized as depressed (23).

### 2.5 Statistical analyses

Kruskal-Wallis test was used to compare ordered categorical data and the chi-square test was used to compare unordered categorical data among Chinese older adults grouped with different numbers of natural teeth. Firstly, we assessed trends in the association between the three dietary patterns and the existing natural teeth separately using a restricted cubic spline. Secondly, the independent effects of the three dietary patterns on the existing natural teeth were analyzed using multiple linear regression, and the results were described using *β* and 95% confidence intervals (CIs). Model I adjusted for the confounding effects of general demographic characteristics and behavioral lifestyle, and Model II further adjusted for the effect of health status. *P* for trend was obtained by analyzing the dietary pattern variables as continuous variables. In addition, to verify the robustness of the results, stratified analyses were conducted for age, sex, residence, education, body type, sleep duration, drinking status, smoking status, and current exercise. The *P* for interaction was obtained by comparing the likelihood ratios of the terms with and without the multiplicative interaction (22).

To further validate the consistency of the association, several sensitivity analyses were conducted. Firstly, to control for confounding by health factors, we restricted participants to those with good self-rated health, no chronic diseases, normal cognition, normal daily activities, and no depression. Secondly, we additionally controlled for confounding by denture wear, brushing frequency, and toothache in fully adjusted models and reassessed associations. Thirdly, we excluded older adults over 105 years of age based on previous studies (24, 37) and reassessed associations. Finally, to minimize recall bias in the super-elderly, we reassessed associations after excluding those aged ≥90 years. Furthermore, we explored the mediating effects of BMI between dietary patterns and extant teeth using a mediating effects analysis tool developed by Andrew F. Hayes (38).

All results were analyzed using SPSS version 26 and R software version 4.4.1.^1^ A two-sided *p* < 0.05 was considered a statistically significant difference.

## 3 Results

Of the general demographic characteristics of the 11,608 participants, 55.96% were female, 38.09% were over 90 years of age, 42.13% lived in rural areas, 70.34% were of general economic status, 49.01% were illiterate, 54.93% were widowed, and 68.61% self-reported a good quality of life.

### 3.1 Basic characteristics of participants according to natural tooth counts

In the results of the quartile grouping of the existing natural teeth, compared to the population in the low existing teeth group (Q1 and Q2), the population in the high existing teeth group (Q3 and Q4) had lower proportions of younger age, female, poor economic status, illiterate, widowed, cognitively impaired, impaired daily activities, and depression; additionally, there were higher proportions of living in city, sleeping 7–8 h, current drinking, current smoking, current exercise, overweight or obese, good self-rated health, and multimorbidity (all *p* < 0.05; Table 1).

**Table 1:** Basic characteristics of participants by natural tooth counts (*n* = 11,608).

Variables	Existing natural teeth					*χ^2^* / *H*	*p*
	Total (*n* = 11,608)	Q1 (*n* = 3,695)	Q2 (*n* = 2,348)	Q3 (*n* = 3,090)	Q4 (*n* = 2,475)		
Female, *n* (%)	6,496 (55.96)	2,301 (62.27)	1,379 (58.73)	1,619 (52.39)	1,197 (48.36)	140.96	**<0.001**
Age, years, *n* (%)						2614.20	**<0.001**
<80	4,327 (37.28)	621 (16.81)	552 (23.51)	1,405 (45.47)	1749 (70.67)		
<90	2,859 (24.63)	836 (22.63)	638 (27.17)	908 (29.39)	477 (19.27)		
≥90	4,422 (38.09)	2,238 (60.57)	1,158 (49.32)	777 (25.15)	249 (10.06)		
Residence, *n* (%)						145.71	**<0.001**
City	2,857 (24.61)	756 (20.46)	491 (20.91)	804 (26.02)	806 (32.57)		
Town	3,861 (33.26)	1,247 (33.75)	850 (36.20)	1,011 (32.72)	753 (30.42)		
Rural	4,890 (42.13)	1,692 (45.79)	1,007 (42.89)	1,275 (41.26)	916 (37.01)		
Economic status, *n* (%)						36.41	**<0.001**
Rich	2,278 (19.62)	713 (19.30)	408 (17.38)	595 (19.26)	562 (22.71)		
General	8,165 (70.34)	2,578 (69.77)	1,686 (71.81)	2,181 (70.58)	1720 (69.49)		
Poor	1,165 (10.04)	404 (10.93)	254 (10.82)	314 (10.16)	193 (7.80)		
Education, *n* (%)						1076.78	**<0.001**
Illiterate	5,689 (49.01)	2,302 (62.30)	1,391 (59.24)	1,315 (42.56)	681 (27.52)		
Primary school	3,709 (31.95)	1,013 (27.42)	680 (28.96)	1,095 (35.44)	921 (37.21)		
Middle school or above	2,210 (19.04)	380 (10.28)	277 (11.80)	680 (22.01)	873 (35.27)		
Widowed, *n* (%)	6,376 (54.93)	2,642 (71.50)	1,517 (64.61)	1,456 (47.12)	761 (30.75)	1159.48	**<0.001**
Self-reported quality of life, *n* (%)						12.12	0.059
Good	7,963 (68.61)	2,518 (68.15)	1,571 (66.94)	2,146 (69.45)	1728 (69.85)		
Fair	3,278 (28.24)	1,058 (28.63)	688 (29.31)	844 (27.31)	688 (27.81)		
Poor	365 (3.14)	119 (3.22)	88 (3.75)	100 (3.24)	58 (2.34)		
Sleep duration, h, *n* (%)						206.21	**<0.001**
<7	4,312 (37.15)	1,302 (35.24)	872 (37.14)	1,216 (39.35)	922 (37.25)		
7–8	4,187 (36.07)	1,174 (31.77)	772 (32.88)	1,162 (37.61)	1,079 (43.60)		
>8	3,109 (26.78)	1,219 (32.99)	704 (29.98)	712 (23.04)	474 (19.15)		
Drinking status, *n* (%)						77.93	**<0.001**
Current	1,655 (14.26)	433 (11.72)	291 (12.39)	478 (15.47)	453 (18.30)		
Former	1,351 (11.64)	383 (10.37)	291 (12.39)	372 (12.04)	305 (12.32)		
Never	8,602 (74.10)	2,879 (77.92)	1766 (75.21)	2,240 (72.49)	1717 (69.37)		
Smoking status, *n* (%)						32.44	**<0.001**
Current	1722 (14.83)	481 (13.02)	326 (13.88)	493 (15.95)	422 (17.05)		
Former	1733 (14.93)	521 (14.10)	359 (15.29)	496 (16.05)	357 (14.42)		
Never	8,153 (70.24)	2,693 (72.88)	1,663 (70.83)	2,101 (67.99)	1,696 (68.53)		
Current exercise, *n* (%)	3,736 (32.18)	875 (23.68)	659 (28.07)	1,089 (35.24)	1,113 (44.97)	339.27	**<0.001**
Body type, *n* (%)						556.75	**<0.001**
Underweight	2,164 (18.64)	969 (26.22)	517 (22.02)	470 (15.21)	208 (8.40)		
Normal	5,673 (48.87)	1826 (49.42)	1,188 (50.60)	1,540 (49.84)	1,119 (45.21)		
Overweight	2,674 (23.04)	601 (16.27)	443 (18.87)	800 (25.89)	830 (33.54)		
Obese	1,097 (9.45)	299 (8.09)	200 (8.52)	280 (9.06)	318 (12.85)		
Self-rated health, *n* (%)						28.22	**<0.001**
Good	5,341 (46.01)	1,687 (45.66)	1,025 (43.65)	1,388 (44.92)	1,241 (50.14)		
Fair	4,645 (40.02)	1,496 (40.49)	966 (41.14)	1,244 (40.26)	939 (37.94)		
Poor	1,622 (13.97)	512 (13.86)	357 (15.20)	458 (14.82)	295 (11.92)		
Cognitive impairment, *n* (%)	2,577 (22.20)	1,263 (34.18)	629 (26.79)	471 (15.24)	214 (8.65)	685.56	**<0.001**
Impaired daily activities, *n* (%)	3,078 (26.52)	1,446 (39.13)	746 (31.77)	608 (19.68)	278 (11.23)	706.10	**<0.001**
Multimorbidity, *n* (%)	7,197 (62.00)	2088 (56.51)	1,416 (60.31)	2010 (65.05)	1,683 (68.00)	100.16	**<0.001**
Depression, *n* (%)	1917 (16.51)	692 (18.73)	429 (18.27)	515 (16.67)	281 (11.35)	66.25	**<0.001**

Existing natural teeth was divided into four groups using quartiles (Q1 0, Q2 1–6, Q3 7–20, Q4 > 20). The Chi-square test was used for comparing unordered categorical data and the Kruskal-Wallis test was used for ordinal data, and bold values indicated statistical significance *p <* 0.05.

### 3.2 Association of dietary patterns with the existing natural teeth

**Figure 2:**
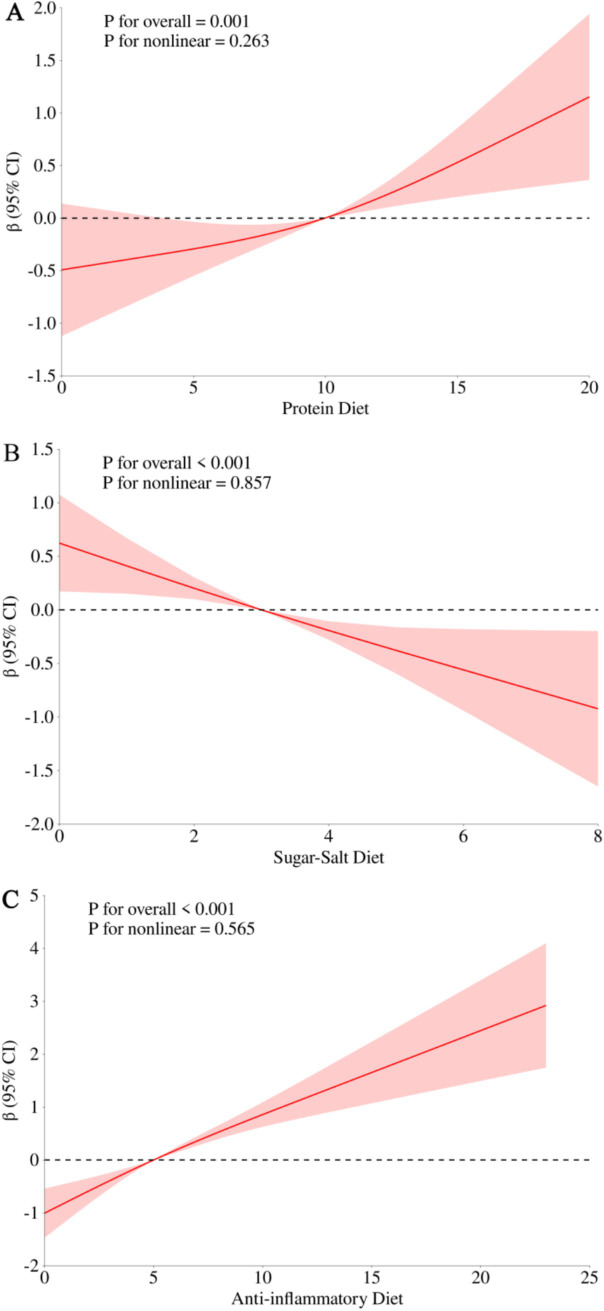
Restricted cubic spline for the association of protein diet, sugar-salt diet, and anti-inflammatory diet with the existing natural teeth. **(A)** Protein diet with existing natural teeth; **(B)** sugar-salt diet with existing natural teeth; **(C)** anti-inflammatory diet with existing natural teeth. Adjusted for sex (male, female), age (<80, <90, ≥90), residence (city, town, rural), economic status (good, general, poor), education (illiterate, primary school, middle school or above), widowed (yes, no), self-reported quality of life (good, fair, poor), sleep duration (<7 h, 7–8 h, >8 h), drinking status (current, former, never), smoking status (current, former, never), current exercise (yes, no), body type (underweight, normal, overweight, obese), self-rated health (good, fair, poor), cognitive function (normal, impaired), activities of daily living (normal, impaired), multimorbidity (yes, no), depression (yes, no).

We used multifactor-adjusted restricted cubic spline to assess the associations of protein diet, sugar-salt diet, and anti-inflammatory diet with the existing natural teeth. Figure 2 showed that the protein diet (*P* for overall = 0.001, *P* for nonlinear = 0.263), sugar-salt diet (*P* for overall <0.001, *P* for nonlinear = 0.857), and anti-inflammatory diet (*P* for overall <0.001, *P* for nonlinear = 0.565) were all linearly associated with the existing natural teeth.

Table 2 showed the associations of protein diet, sugar-salt diet, and anti-inflammatory diet with the existing natural teeth. In a fully adjusted model of potential confounders (Model II), we found that the high protein diet (Q4) had the most teeth retention (*β*: 1.70, 95% CI: 1.14, 2.25; *P* for trend <0.001) compared to the low protein diet (Q1); the high sugar-salt diet (Q4) had the least tooth retention (*β*: −1.14, 95%CI: −1.61, −0.67; *P* for trend <0.001) compared to the low sugar-salt diet (Q1); the high anti-inflammatory diet (Q4) had the most teeth retention (*β*: 1.98, 95%CI: 1.45, 2.51; *P* for trend <0.001) compared to the low anti-inflammatory diet (Q1).

**Table 2:** Association of protein diet, sugar-salt diet, and anti-inflammatory diet with the existing natural teeth.

Variables	n	β (95%CI)		
		Crude model	Model I	Model II
Protein diet				
Q1	3,080	0.00 (Reference)	0.00 (Reference)	0.00 (Reference)
Q2	2,880	**2.06 (1.53, 2.59)**	**0.53 (0.06, 0.99)**	**0.48 (0.01, 0.94)**
Q3	3,225	**3.48 (2.96, 3.99)**	**1.11 (0.64, 1.58)**	**1.06 (0.59, 1.52)**
Q4	2,423	**5.11 (4.56, 5.66)**	**1.74 (1.19, 2.30)**	**1.70 (1.14, 2.25)**
*P* for trend		**<0.001**	**<0.001**	**<0.001**
Sugar-salt diet				
Q1	3,094	0.00 (Reference)	0.00 (Reference)	0.00 (Reference)
Q2	3,303	**−0.61 (−1.12, −0.09)**	**−0.64 (−1.09, −0.20)**	**−0.61 (−1.06, −0.17)**
Q3	2,305	**−1.25 (−1.82, −0.68)**	**−0.79 (−1.28, −0.30)**	**−0.83 (−1.32, −0.34)**
Q4	2,906	**−1.12 (−1.65, −0.59)**	**−1.10 (−1.57, −0.63)**	**−1.14 (−1.61, −0.67)**
*P* for trend		**<0.001**	**<0.001**	**<0.001**
Anti-inflammatory diet				
Q1	3,091	0.00 (Reference)	0.00 (Reference)	0.00 (Reference)
Q2	3,186	**1.36 (0.85, 1.87)**	0.45 (−0.01, 0.91)	0.40 (−0.05, 0.86)
Q3	2,475	**2.71 (2.16, 3.25)**	**0.96 (0.46, 1.46)**	**0.88 (0.38, 1.38)**
Q4	2,856	**5.76 (5.23, 6.28)**	**2.08 (1.55, 2.61)**	**1.98 (1.45, 2.51)**
*P* for trend		**<0.001**	**<0.001**	**<0.001**

Model I was adjusted for sex (male, female), age (<80, <90, ≥90), residence (city, town, rural), economic status (good, general, poor), education (illiterate, primary school, middle school or above), widowed (yes, no), self-reported quality of life (good, fair, poor), sleep duration (<7 h, 7–8 h, >8 h), drinking status (current, former, never), smoking status (current, former, never), current exercise (yes, no). Model II was further adjusted for body type (underweight, normal, overweight, obese), self-rated health (good, fair, poor), cognitive function (normal, impaired), activities of daily living (normal, impaired), multimorbidity (yes, no), depression (yes, no). Bold values indicated statistical significance *p* < 0.05.

Further, we regrouped the existing teeth (<20, ≥20) and reassessed the associations. Supplementary Table S2 showed that in a fully adjusted model with potential confounders, a 28% lower risk of fewer than 20 teeth (OR: 0.72, 95%CI: 0.61, 0.84; *P* for trend <0.001) for the high-protein diet (Q4) compared with the low-protein diet (Q1); a risk of fewer than 20 teeth was increased by 44% (OR: 1.44, 95%CI: 1.26, 1.64; *P* for trend <0.001) with the high sugar-salt diet (Q4) compared with the low sugar-salt diet (Q1); a 38% lower risk of fewer than 20 tooth (OR: 0.62, 95%CI: 0.53, 0.72; *P* for trend <0.001) for the high anti-inflammatory diet (Q4) compared with the low anti-inflammatory diet (Q1).

### 3.3 Stratification and sensitivity analysis

**Figure 3:**
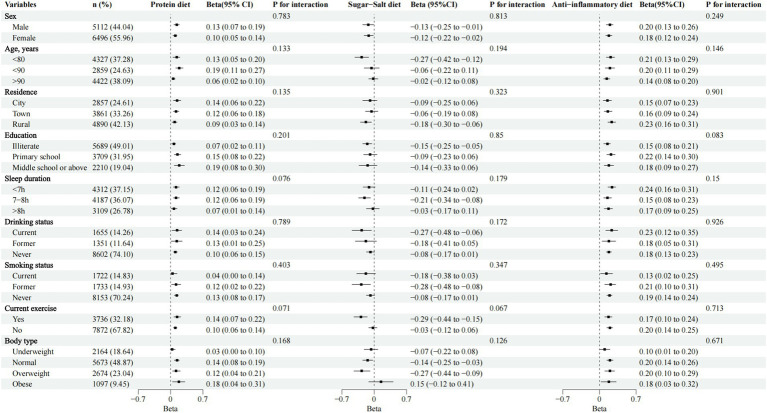
Association between protein diet, sugar-salt diet, and anti-inflammatory diet with the existing natural teeth, stratification analyses. Adjusted for sex (male, female), age (<80, <90, ≥90), residence (city, town, rural), economic status (good, general, poor), education (illiterate, primary school, middle school or above), widowed (yes, no), self-reported quality of life (good, fair, poor), sleep duration (<7 h, 7–8 h, >8 h), drinking status (current, former, never), smoking status (current, former, never), current exercise (yes, no), body type (underweight, normal, overweight, obese), self-rated health (good, fair, poor), cognitive function (normal, impaired), activities of daily living (normal, impaired), multimorbidity (yes, no), depression (yes, no).

In stratified analyses, the associations between dietary patterns and the existing natural teeth were consistent across subgroups of age, sex, residence, education, body type, sleep duration, drinking status, smoking status, and current exercise, with *P* for interaction >0.05 (Figure 3). Meanwhile, we conducted several sensitivity analyses. Firstly, the associations between dietary patterns and the existing natural teeth were robust when we restricted participants to those who had good self-rated health, no chronic diseases, normal cognition, normal daily activities, and no depression. Second, we additionally controlled for denture wear, brushing frequency, and toothache and found consistent results. Finally, the associations between dietary patterns and the existing natural teeth remained robust after those extremely old participants were excluded (Supplementary Tables S3, S4).

### 3.4 Joint effects of protein diet, sugar-salt diet, and anti-inflammatory diet on existing natural teeth

We explored the joint effect of a protein diet, sugar-salt diet, and anti-inflammatory diet on existing natural teeth after grouping them by the median. Using those with diets high in protein, low in sugar-salt, and high in anti-inflammatory as the reference group, we found that most of the other groups had lower existing natural teeth, with those with diets low in protein, high in sugar-salt, and low in anti-inflammatory diets having the lowest existing teeth (*β*: −1.97, 95% CI: −2.61, −1.33; Table 3).

**Table 3:** Joint effect of protein diet, sugar-salt diet, and anti-inflammatory diet on existing natural teeth.

Protein diet	Sugar-Salt diet	Anti-inflammatory diet	n	β (95%CI)
≤ 10	> 3	≤ 5	1,683	**−1.97 (−2.61, −1.33)**
		> 5	764	**−1.65 (−2.43, −0.87)**
	≤ 3	≤ 5	2,698	**−1.39 (−1.97, −0.80)**
		> 5	815	0.08 (−0.68, 0.84)
> 10	> 3	≤ 5	798	**−0.99 (−1.75, −0.22)**
		> 5	1966	−0.31 (−0.88, 0.27)
	≤ 3	≤ 5	1,098	**−0.75 (−1.43, −0.06)**
		> 5	1786	0.00 (Reference)

Protein diet, sugar-salt diet, and anti-inflammatory diet were grouped according to median. Adjusted for sex (male, female), age (<80, <90, ≥90), residence (city, town, rural), economic status (good, general, poor), education (illiterate, primary school, middle school or above), widowed (yes, no), self-reported quality of life (good, fair, poor), sleep duration (<7 h, 7–8 h, >8 h), drinking status (current, former, never), smoking status (current, former, never), current exercise (yes, no), body type (underweight, normal, overweight, obese), self-rated health (good, fair, poor), cognitive function (normal, impaired), activities of daily living (normal, impaired), multimorbidity (yes, no), depression (yes, no). Bold values indicated statistical significance *p* < 0.05.

### 3.5 Mediation analysis of BMI between dietary patterns and existing natural teeth

**Figure 4:**
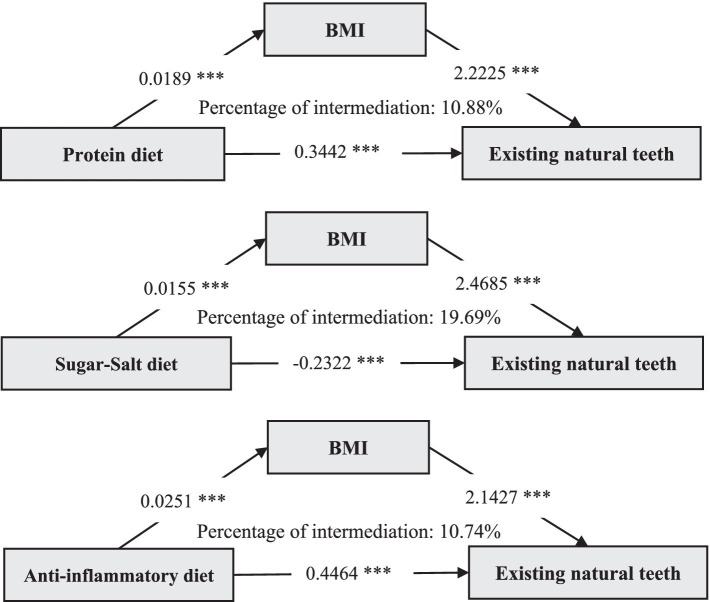
Mediating pathway effects of BMI on the association between dietary patterns and existing natural teeth. BMI, Body Mass Index; *p* < 0.001***.

We analyzed the mediating role of BMI on the association of dietary patterns with extant natural teeth, and the path model for the mediation analysis was shown in Figure 4. We found that a higher protein diet, sugar-salt diet, and anti-inflammatory diet were all associated with increased BMI (Supplementary Figure S1; *p* < 0.001), and high BMI was associated with a higher number of teeth. Further findings showed that BMI was associated with a relative mediating effect ratio of 10.88% for the protein diet with existing natural teeth, 19.69% for the sugar-salt diet with existing natural teeth, and 10.74% for the anti-inflammatory diet with existing natural teeth (Figure 4).

## 4 Discussion

### 4.1 Main findings

In the 2017–2019 Chinese Community Study of Chinese aged 65 years and older, we found that protein diet, sugar-salt diet, and anti-inflammatory diet at around age 60 years were all linearly associated with existing natural teeth. Higher protein and anti-inflammatory diets were associated with less tooth loss (28% and 38% risk decrease for fewer than 20 teeth, respectively), and higher sugar-salt diet was associated with more tooth loss (44% risk increase for fewer than 20 teeth). We further found a joint effect of low protein, high sugar-salt, and low anti-inflammatory diets on tooth loss. Moreover, BMI mediated 10.88%, 19.69%, and 10.74% of the mediating effects between the three dietary patterns and existing natural teeth, respectively. To the best of our knowledge, this is the first study in China to explore the association between multiple dietary patterns and existing natural teeth in a community-based elderly population.

### 4.2 Comparison with other studies

The association between dietary patterns and oral health has been extensively studied (39), with findings that support the results of our study. Prior research has consistently demonstrated that high-protein diets are beneficial for oral health, particularly for preserving alveolar bone and existing natural teeth (17, 40). Swedish scholars showed that protein diets such as fish were positively associated with existing teeth (*β* = 0.19, *p* = 0.001) (41). Studies in Japan have highlighted that protein-rich diets (Fish/meat) not only contribute to maintaining overall bone density but also reduce the risk of periodontal disease and subsequent tooth loss by supporting collagen synthesis and tissue repair (OR: 1.14, 95%CI: 1.07, 1.22) (42, 43). Our findings align with these studies and further confirm their relevance in the Chinese elderly population. In contrast, the deleterious effects of high sugar and salt consumption on oral health are well-documented (14). Excessive sugar intake, a major risk factor for dental caries, promotes the proliferation of cariogenic bacteria such as *Streptococcus mutans*, leading to enamel demineralization and tooth decay (44, 45). The World Health Organization has similarly advocated that sugar should be limited to no more than 5% of energy intake (46). A study from Norway showed that dessert diets increase the risk of tooth loss (≤19 teeth) by 2.52 times (47). Meanwhile, high salt intake has been associated with hypertension and systemic inflammation (48), which may exacerbate periodontal diseases indirectly (49, 50). Our study builds on these findings by demonstrating the compounded risks of a sugar-salt dietary pattern, particularly in rural areas with salt-pickled vegetables and sugary dessert diets. The anti-inflammatory dietary pattern has also been recognized as a key factor in mitigating chronic inflammatory conditions, including periodontal disease (51). Previous studies have shown that diets rich in fruits, vegetables, and polyunsaturated fatty acids—common components of anti-inflammatory diets—can reduce levels of systemic inflammatory markers such as C-reactive protein (CRP) and interleukin-6 (IL-6) (52). By controlling inflammation, these diets likely prevent periodontal destruction (15), which is a major cause of tooth loss in the elderly. Our findings extend this evidence by quantifying the reduced risk of significant tooth loss (<20 teeth) associated with higher adherence to anti-inflammatory diets.

Notably, previous research has often focused on isolated nutrients or dietary components (17, 18), while our study emphasizes the holistic impact of broader dietary patterns. This approach aligns with contemporary nutritional epidemiology, which recognizes that dietary components interact synergistically rather than acting in isolation (52). Furthermore, the consistency of our results across subgroups defined by age, sex, residence, and other demographic factors suggests that these associations are robust and broadly applicable, regardless of cultural or socioeconomic contexts. It is also important to highlight the novel findings of our study compared to existing literature. Few previous studies have examined the joint effects of multiple dietary patterns on tooth retention. Our results reveal a compounded risk when unfavorable dietary habits, such as low protein intake, high sugar-salt intake, and low anti-inflammatory dietary adherence, coexist. This synergistic interaction emphasizes the need for multi-faceted dietary interventions to improve oral health outcomes.

Additionally, the mediation analysis of BMI provides insights into the metabolic pathways linking diet and existing natural teeth. While earlier studies by Swedish and Israeli scholars have recognized obesity as a risk factor for periodontal disease and tooth loss (20, 21), our results suggest otherwise. It is difficult to accept that overweight or obesity in the elderly population protects the number of existing teeth, but the “obesity paradox” that people with high BMI may exhibit certain health advantages has been proposed and explained in the literature (53). Older adults with a high BMI may have a richer diet, particularly in terms of intake of nutrients associated with dental health, such as protein, calcium, vitamin D, and vitamin C, resulting in enhanced generalized bone density (54). Also, leptin and adiponectin, which are released by adipose tissue at low concentrations, may have anti-inflammatory and immune-promoting properties that help slow inflammatory destruction of periodontal tissue (55). On the other hand, overweight or obese people may also prompt more frequent oral hygiene management, such as regular checkups and cleanings, indirectly protecting the teeth (56).

### 4.3 Strengths and limitations

This study has several strengths that enhance the reliability and applicability of our findings. First, the large-scale, community-based cohort provided a robust dataset that allowed for comprehensive adjustment of potential confounders. Second, our focus on multiple dietary patterns, rather than isolated nutrients, reflects real-world dietary behaviors and enables broader insights into dietary interventions. Third, the incorporation of advanced statistical methods, such as restricted cubic spline models and mediation analysis, ensures a nuanced understanding of the associations and mechanisms involved. Lastly, the consistency of results across subgroups and in sensitivity analyses strengthens the generalizability of our findings.

However, several limitations warrant consideration. First, the cross-sectional design limits the ability to make causal inferences, and prospective cohort studies are needed to confirm the temporal relationships. Second, although we explored oral health effects using dietary frequency in older adults around age 60 years, self-reported methods of assessing dietary intake are subject to recall bias and may not capture seasonal dietary changes. Third, the study population was limited to older adults in China, which may limit the generalizability to other age groups or ethnic populations. Fourth, while we adjusted for many confounders, residual confounders (e.g., fatigue, oral microbes, genetics) could not be completely excluded.

Future studies should explore the longitudinal effects of dietary patterns on existing teeth to determine causality. In addition, techniques such as microbiomics and metabolomics can provide more insight into the biological pathways between diet and oral health, especially in the context of microbiome-diet interactions. Finally, expanding the scope of the study to include different populations and younger age groups could provide a more comprehensive understanding of the impact of diet on oral health across the life course.

## 5 Conclusion

Our results suggested that increasing the frequency of protein and anti-inflammatory diets while reducing sugar and salt intake may be an effective strategy for improving the number of extant teeth in older adults. The mediating effect of BMI indicated the dual benefits of a rational diet targeting both body type health and the number of teeth retained. Our study both enriched the use of rational diets in health outcomes and provided new perspectives on oral health. Future dietary guidelines for older adults should consider including recommendations that explicitly address oral health. Nutritionists, dentists, and public health practitioners should increase interdisciplinary collaboration to develop comprehensive strategies aimed at improving dietary habits, body type, and reducing tooth loss in the aging population.

## Data Availability

The datasets presented in this study can be found in online repositories. The names of the repository/repositories and accession number(s) can be found at: The publicly available Chinese Longitudinal Healthy Longevity Survey (https://opendata.pku.edu.cn/dataverse/CHADS).

## Footnotes

^1^ http://www.R-project.org
