# Supplementary material for: Association between dietary patterns and existing natural teeth in Chinese elderly: a national community-based study
Source: Front Nutr. 2025 Mar 13;12:1549181. doi: 10.3389/fnut.2025.1549181 (PMC11966427; doi:10.3389/fnut.2025.1549181)

Supplementary Table S1. Characteristics of the population with missing covariates

**Variables**	**Included (n=11608)**	**Excluded (n=1072)**	**P-value**
Number of natural teeth, Mean±SD	10.0±10.6	9.84±8.24	0.252
Protein diet, Mean±SD	9.97±5.04	9.47±5.44	0.769
Anti-inflammatory diet, Mean±SD	5.75±4.48	5.77±4.31	0.578
Sugar-Salt diet, Mean±SD	3.12±2.16	3.08±2.17	0.664

Supplementary Table S2. Logistic regression for regrouping the number of natural teeth (<20, ≥20)

Variables	n	OR (95%CI)		
		**Crude model**	**Model I**	**Model II**
**Protein diet**				
Q1	3080	1.00 (Reference)	1.00 (Reference)	1.00 (Reference)
Q2	2880	**0.69 (0.61 ~ 0.78)**	0.93 (0.81 ~ 1.07)	0.95 (0.82 ~ 1.09)
Q3	3225	**0.53 (0.47 ~ 0.60)**	**0.81 (0.71 ~ 0.93)**	**0.83 (0.72 ~ 0.95)**
Q4	2423	**0.40 (0.35 ~ 0.45)**	**0.71 (0.60 ~ 0.83)**	**0.72 (0.61 ~ 0.84)**
P for trend		<0.001	<0.001	<0.001
**Sugar-Salt diet**				
Q1	3094	1.00 (Reference)	1.00 (Reference)	1.00 (Reference)
Q2	3303	**1.16 (1.04 ~ 1.30)**	**1.22 (1.08 ~ 1.38)**	**1.21 (1.07 ~ 1.37)**
Q3	2305	**1.30 (1.15 ~ 1.46)**	**1.25 (1.09 ~ 1.44)**	**1.27 (1.11 ~ 1.46)**
Q4	2906	**1.32 (1.18 ~ 1.48)**	**1.42 (1.24 ~ 1.62)**	**1.44 (1.26 ~ 1.64)**
P for trend		<0.001	<0.001	<0.001
**Anti-inflammatory diet**				
Q1	3091	1.00 (Reference)	1.00 (Reference)	1.00 (Reference)
Q2	3186	**0.73 (0.65 ~ 0.83)**	**0.86 (0.75 ~ 0.98)**	**0.87 (0.76 ~ 0.99)**
Q3	2475	**0.57 (0.50 ~ 0.65)**	**0.76 (0.66 ~ 0.88)**	**0.78 (0.67 ~ 0.90)**
Q4	2856	**0.34 (0.30 ~ 0.38)**	**0.60 (0.52 ~ 0.70)**	**0.62 (0.53 ~ 0.72)**
P for trend		<0.001	<0.001	<0.001

Note: Model I was adjusted for sex (male, female), age (<80, <90, ≥90), residence (city, town, rural), economic status (good, general, poor), education (illiterate, primary school, middle school or above), widowed (yes, no), self-reported quality of life (good, fair, poor), sleep duration (<7 h, 7-8 h, >8 h), drinking status (current, former, never), smoking status (current, former, never), current exercise (yes, no).

Model II was further adjusted for body type (underweight, normal, overweight, obese), self-rated health (good, fair, poor), cognitive function (normal, impaired), activities of daily living (normal, impaired), multimorbidity (yes, no), depression (yes, no).

Supplementary Table S3. Sensitivity analyses of the association between protein diet, sugar-salt diet, and anti-inflammatory diet with the number of natural teeth

Outcomes	n (%)	β (95%CI)		
		Protein diet	Sugar-Salt diet	Anti-inflammatory diet
**[a] 1. Limited population:**				
Good self-rated health	5341 (46.01)	0.09 (0.03, 0.14)	-0.27 (-0.38, -0.15)	0.18 (0.12, 0.24)
No chronic disease	4411 (38.00)	0.08 (0.02, 0.14)	-0.22 (-0.34, -0.10)	0.14 (0.07, 0.21)
Normal cognition	9031 (77.80)	0.13 (0.09, 0.18)	-0.23 (-0.32, -0.14)	0.19 (0.14, 0.24)
Normal daily activities	8530 (73.48)	0.13 (0.08, 0.18)	-0.27 (-0.37, -0.18)	0.19 (0.14, 0.25)
No depression	9691 (83.49)	0.11 (0.07, 0.15)	-0.23 (-0.32, -0.15)	0.18 (0.13, 0.22)
**[b] 2. Additional covariates:**				
	11536 (100)	0.11 (0.08, 0.15)	-0.14 (-0.21, -0.07)	0.17 (0.13, 0.21)
**[a] 3. Exclusion of persons older than 105 years:**				
	11394 (100)	0.12 (0.08 to 0.16)	-0.20 (-0.28 to -0.12)	0.18 (0.14 to 0.23)

[a] Adjusted for sex (male, female), age (<80, <90, ≥90), residence (city, town, rural), economic status (good, general, poor), education (illiterate, primary school, middle school or above), widowed (yes, no), self-reported quality of life (good, fair, poor), sleep duration (<7 h, 7-8 h, >8 h), drinking status (current, former, never), smoking status (current, former, never), current exercise (yes, no), body type (underweight, normal, overweight, obese), self-rated health (good, fair, poor), cognitive function (normal, impaired), activities of daily living (normal, impaired), multimorbidity (yes, no), depression (yes, no).

[b] Further adjusted for denture wear (yes, no), brushing frequency, and toothache (yes, no).

Supplementary Table S4. Associations between protein diet, sugar-salt diet, and anti-inflammatory diet and the number of natural teeth after exclusion of older adults ≥90 years of age

Variables	n	β (95%CI)
Protein diet		
Q1	1607	0.00 (Reference)
Q2	1823	**0.92 (0.24, 1.60)**
Q3	2153	**1.46 (0.79, 2.14)**
Q4	1603	**2.05 (1.25, 2.85)**
P for trend		**<0.001**
Sugar-Salt diet		
Q1	1957	0.00 (Reference)
Q2	2079	**-0.74 (-1.36, -0.11)**
Q3	1375	**-0.91 (-1.61, -0.21)**
Q4	1775	**-1.56 (-2.22, -0.90)**
P for trend		**<0.001**
Anti-inflammatory diet		
Q1	1632	0.00 (Reference)
Q2	1914	0.63 (-0.05, 1.30)
Q3	1570	**1.20 (0.47, 1.92)**
Q4	2070	**2.29 (1.54, 3.03)**
P for trend		**<0.001**

Adjusted for sex (male, female), age (<80, <90, ≥90), residence (city, town, rural), economic status (good, general, poor), education (illiterate, primary school, middle school or above), widowed (yes, no), self-reported quality of life (good, fair, poor), sleep duration (<7 h, 7-8 h, >8 h), drinking status (current, former, never), smoking status (current, former, never), current exercise (yes, no), body type (underweight, normal, overweight, obese), self-rated health (good, fair, poor), cognitive function (normal, impaired), activities of daily living (normal, impaired), multimorbidity (yes, no), depression (yes, no).

Supplementary Figure S1. Restricted Cubic Spline for the association of protein diet, sugar-salt diet, and anti-inflammatory diet with the BMI

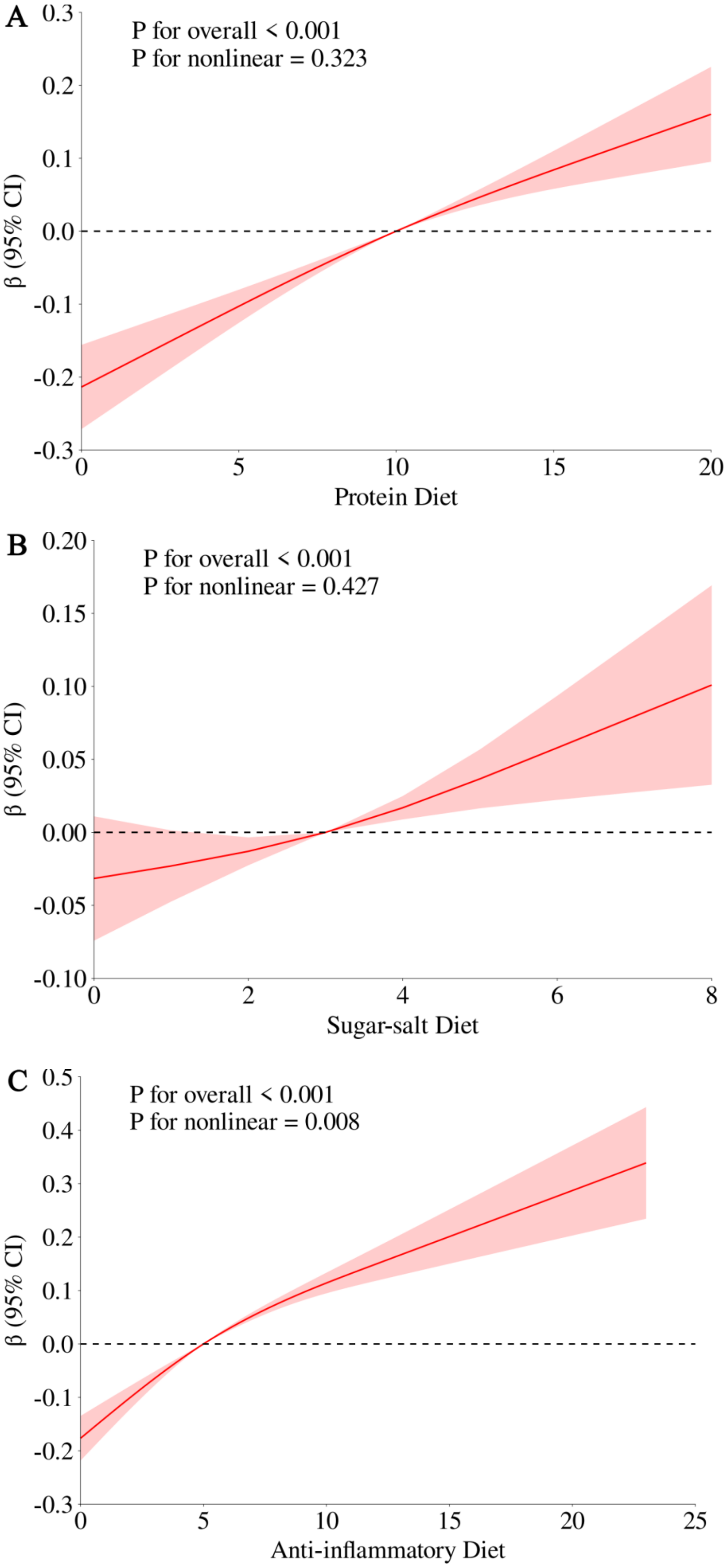

Supplement: Supplementary file 1 [file Data_Sheet_1.pdf]
